# Inter-Individual Differences in Cognitive Response to a Single Bout of Physical Exercise—A Randomized Controlled Cross-Over Study

**DOI:** 10.3390/jcm8081101

**Published:** 2019-07-25

**Authors:** Svenja Schwarck, Marlen Schmicker, Milos Dordevic, Kathrin Rehfeld, Notger Müller, Patrick Müller

**Affiliations:** 1 Neuroprotection Lab, German Center of Neurodegenerative Diseases (DZNE), 39120 Magdeburg, Germany; 2 Institute of Cognitive Neurology and Dementia research, Otto-von-Guericke-University Magdeburg, 39120 Magdeburg, Germany; 3 Institute of Sport Science, Otto-von-Guericke-University Magdeburg, 39104 Magdeburg, Germany; 4 Center for Behavioral Brain Science (CBBS), 39106 Magdeburg, Germany; 5 Medical Faculty, Clinic for Neurology, Otto-von-Guericke-University Magdeburg, 39120 Magdeburg, Germany

**Keywords:** acute exercise, cognition, lactate, responder, personalized medicine

## Abstract

Recent reviews have shown that acute exercise can improve cognitive functions, especially executive functions. However, a closer look at the included studies revealed a wide inter-individual variability in the effects of exercise on cognition. Therefore, thirty-nine healthy adults (age: 19–30 years) were analyzed in a randomized, controlled cross-over study with two exercise groups (*n* = 13 each) and a sedentary control group (*n* = 13). The exercise conditions included moderate (30 min at 40–59% VO_2max_) and high intensity interval (five × 2 min at 90% VO_2max_ with 3 min active recovery at 40% VO_2max_) treadmill exercise. The main outcome assessed was cognitive performance (attention, inhibitory control, cognitive flexibility) and underlying inter-individual variability in young adults. On the group level no significant group or group × time interaction effects were observed. Using a median split, we found significant differences between low and high cognitive performers regarding cognitive function following moderate and high intensity interval treadmill exercise. Furthermore, using a pre-determined threshold we could identify responders and non-responders to acute exercise. Therefore, future research should consider individual performance requirements.

## 1. Introduction

Regular physical exercise is a cost-effective intervention with important health benefits, in particular, reducing the risk of cancer, diabetes, coronary heart and neurodegenerative diseases [1,2,3]. Additionally, physical exercise can enhance cognitive function [4,5]. Even a single bout of physical exercise (acute exercise) has a small but positive effect on cognition with a Cohen’s *d* of 0.097 [6]. The greatest benefits (effect size 0.2–1.16) following a single session of physical exercise have been shown for the performance in the Stroop task assessing inhibitory control as a part of executive functions [7]. On this occasion the largest effects are shown for reaction time while accuracy is unaffected [8]. However, there are fewer studies reporting beneficial effects on other cognitive domains such as attention, memory or other components of executive functions [6]. 

Furthermore, exercise intensity seems to modulate the impact on cognitive performance. A robust body of literature has shown greatest benefits in inhibitory control after a single bout of moderate physical intensity [9,10]. Therefore, an inverted U-shape for the dose–response relationship has been suggested [11], depicting higher performance after moderate but not after very high or very low intensity exercise [8]. In contrast, some studies show a greater cognitive benefit after a single session of high intensity compared to moderate intensity exercise [12,13]. Consequently, this topic requires further investigation.

Moreover, a closer look at the results of these studies reveals a wide inter-individual variability in the effects of several exercise modalities (e.g., moderate or high intensity) on cognition [14]. Most exercise protocols of the studies incorporated in meta-analyses and reviews [6,7] are based on exercise recommendations, such as those from the World Health Organization [15]. Also, the reported positive results mostly reflect the main effects of multivariate analyses. However, within-group inter-individual differences in systemic or cellular adaptation are usually considered as an error, which reduces the effect size [16]. Several studies indicate that the adaptive response in cardiorespiratory fitness within apparently homogenous groups is highly variable to both long-term moderate and high intensity interval training protocols [17,18]. Hence, exercise effects on VO_2max_, insulin sensitivity and other cardiovascular as well as metabolic parameters display large inter-individual heterogeneity. 

An approach of personalised sports medicine is to divide the apparently homogenous groups into responders, who show a positive physical improvement, and non-responders, whose physical performance remains unchanged or even gets worse [19]. In a cross-over design using a 3-week moderate (30 min at 65% VO_2max_) and sprint training (8 × 20 s at 170% VO_2max_ following with each 10 s recovery). Bonafiglia and colleagues [20] found a number of non-responders for each exercise protocol but no global non-responders. A subject who failed to adapt to the moderate exercise protocol showed physical improvements in the sprint protocol or vice versa. 

Currently, there are no studies available which have investigated the inter-individual heterogeneity in the cognitive response to a single bout of physical exercise. Therefore, this study aimed to analyse the effects of a single bout of moderate and high intensity physical exercise regarding potential inter-individual cognitive differences within the framework of responders and non-responders. In this connection, inter-individual differences and the associated responder such as non-responder especially for cognitive tests assessing executive functions were assumed. 

## 2. Experimental Section

### 2.1. Subjects 

The study was designed as a 3-week randomized, controlled cross-over study (registration number: DRKS00017213) and was approved by the ethics committee of the Otto-von-Guericke University, Magdeburg, Germany. All subjects signed a written informed consent form for participation and received payment (50€) and/or course credits. Thirty-nine healthy male students, aged 19–30 years (*M* = 23.33; *SD* = 3.23) were recruited through university courses and postings. The sample in this study was physically active (≤3 sessions a week) with a normal BMI (*M* = 24.34; *SD* = 1.91). All participants were right-hand dominant and free of any cardiovascular, neurological (Beck Depression Inventory Revision II; BDI-II < 11) and pulmonary disorder, colour-blindness and uncorrected vision. All participants were asked to maintain their regular physical activity and nutritional habits. 

### 2.2. Experimental Design

**Figure 1:**
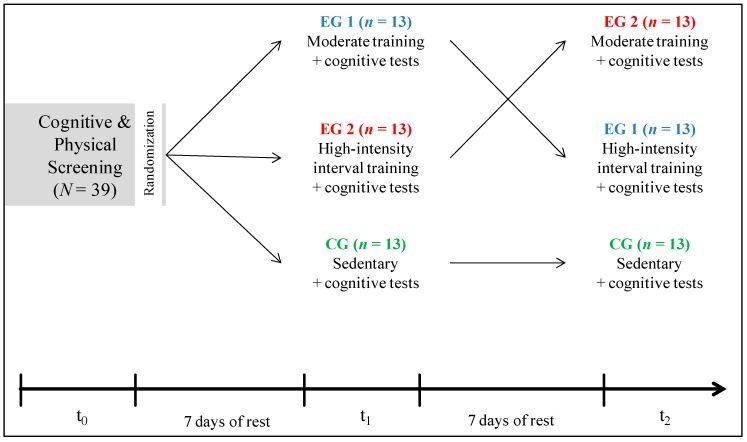
Experimental design. *N* = 39. Study included three sessions (t0, t1, t2) and three groups: experimental group 1 (EG1, *n* = 13), experimental group 2 (EG2, *n* = 13) and control group (CG, *n* = 13). The experimental groups performed either a single bout of moderate intensity or a high-intensity interval training (hiit) while the control group was sedentary. Main outcome variable was cognitive performance.

The study included three sessions separated each by seven days (Figure 1). First, all subjects completed a cognitive test battery (baseline comparison) in a screening session (t0) and were tested for their cardiovascular fitness in order to receive comparable levels for each individual’s exercise intensity. Using a crossover design, subjects were randomly assigned into three groups (each *n* = 13). Experimental group 1 (EG1) started with a single bout of moderate physical exercise on t1 and with a high intensity interval training (hiit) on t2. The experimental group 2 (EG2) performed the physical exercise vice versa. The inactive control group (CG) was sitting sedentary for 10 min before measurements. The main cognitive outcome measurements comprised attention, inhibitory control and cognitive flexibility. All measurements were conducted at the same time of the day for each subject to control circadian rhythm. Each participant abstained from food and caffeine at least two hours before the testing started. All participants also refrained from any physical activity and the consumption of alcohol in the 24 hours before each session.

#### 2.2.1. Screening (t0)—Incremental Step Test

In the screening session all subjects completed an incremental step test to voluntary exhaustion to determine their individual VO_2max_ on a treadmill (HCP Cosmos Pulsar 3 P, Germany). To ensure safety, all subjects used a safety harness which stops the treadmill in case of an emergency. Warming up included a three-minute walk at 4 km/h. The velocity increased 2 km/h every three minutes. During the incremental step test, breath-by-breath pulmonary gas-exchange data (MetaSoft^®^ Studio: Cortex Biophysik GmbH Leipzig, Germany) and heart rate (Polar: H7 heart rate sensor, Finland) were assessed. To determine metabolic status, capillary blood samples were taken from the ear at rest (baseline), after each step and one, three and five minutes after the step test was finished. The lactate concentration was measured with lactate scout sensors (SensLab GmbH Leipzig, Germany). Ratings of perceived exertion were collected after each lactate assessment using a 6–20 Borg Scale [21]. The incremental step test was completed when (i) the respiratory exchange ratio was above 1.10, (ii) a plateau in VO_2_ occurred (despite increasing workload) or (iii) the subject stopped running. 

#### 2.2.2. Exercise Conditions (t1, t2)

**Figure 2:**
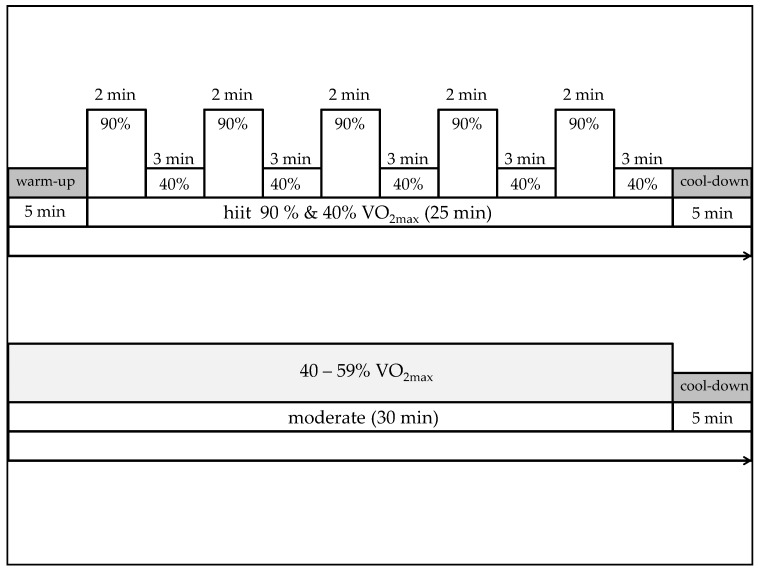
Both physical exercise conditions lasted 35 min each. Single session physical exercise in moderate intensity: 30 min at 40–59% VO_2max_ and 5 min warm up at 40% VO_2max_. Single session high intensity interval training (hiit): 5 min warm up at 40% VO_2max_, 5 two-minute periods at 90% VO_2max_ each followed by three-minute recovery periods at 40% VO_2max_ and a cool down at 40% VO_2max_.

All subjects of the experimental group performed a single session of physical exercise wearing a safety harness on a treadmill. Heart rate, metabolic status and perceived exertion were continuously assessed during the sessions. Both single bouts of physical exercise lasted 35 min (Figure 2). The moderate session included 30 min at 40–59% VO_2max_ followed by a five-minute cool down at 40% VO_2max_. Lactate samples and Borg rating were collected at baseline, after 30 min and at the end of the session. The high intensity interval training consisted of a five-minute warm up at 40% VO_2max_, 5 two-minute periods at 90% VO_2max_ each followed by three-minute recovery periods at 40% VO_2max_ and a cool down at 40% VO_2max_. Lactate samples and Borg rating were assessed at baseline, after the warm up, after the whole interval and at the end of the physical exercise. 

#### 2.2.3. Neuropsychological Measures

Neuropsychological measurements included the domains attention (d2-R) and executive functions (Stroop task, Trail Making Task) and were performed 10 min after each acute exercise (EG) or sitting (CG) condition. 

The speeded paper-pencil task d2-R by Brickenkamp et al. [22] assesses the concentration and selective attention. The task required to cross all targets (*d* with two dashes) surrounded by distractors as soon and accurate as possible (14 lines, each 20 s). The main outcome was the total number of responses (BZO) and the number of all errors related to the total number of responses (F%).

Stroop task and Trail Making Task (TMT) were assessed with standardized and validated computer-based Wiener Testsystem [23]. The main outcome assessed is reaction time. The Stroop task was integrated to examine the inhibitory control. The test contains two separated conditions: reading and naming. Each condition included a congruent task (meaning and colour match) and an incongruent task (meaning and colour differ), for example, the word “blue” printed in red colour. The Stroop interference effect (reaction time congruent – reaction time incongruent) was calculated for each condition. The Trail Making Task (TMT) consists of two parts. Subjects were asked to connect numbered cycles (TMT-A) or numbers and letters (TMT-B) in ascending order. The first part reflects the information processing speed and the second part measures the cognitive flexibility. 

### 2.3. Statistical Analysis 

On group level, the demographic data were analysed using a one-way ANOVA. Moreover, the variables BZO and F% (d2-R), reading and naming interference (Stroop task) and part A and B such as the ratio of B/A (TMT) were analyzed using a two-way repeated measures ANOVA after normal distribution was confirmed. The factors were group (EG1, EG2, CG) and time (t0, t1, t2). Additionally, using a median split all participants in the two experimental groups were divided into low and high cognitive performers for cognitive composite score and each cognitive variable and associated intensity condition (each *n* = 13). The groups (high cognitive performance, low cognitive performance, CG, each *n* = 13) were analyzed using a one-way ANOVA. Specific differences were identified using Bonferroni or Games-Howell post-hoc tests. All multiple comparisons were Bonferroni corrected. The statistically significant level was defined at *p* < 0.05. For the verification of inter-individual analysis, the change score of the control group (resting condition–t0; Variance 1) and the exercise group (exercise condition–t0, Variance 2) was calculated for each test variable. Thereafter, Levene’s test was run to detect potential differences regarding the change score of the variances of both groups (Variance 1 vs. Variance 2). Only test variables with significant differences (indicator of additional variability in the exercise group) were included for further analysis. For individual analysis, the confidence interval (CI) combined with the smallest worthwhile change (SWC)—a pre-determined threshold for the smallest meaningful change score after an intervention—was applied. Therefore, the SCW for each acute exercise condition (0.2 × between-subject standard deviation) and the associated 50% CI (post-pre score change ± typical error) for each subject was calculated. Subsequently, for each exercise condition participants were divided into responder (true score change CI above SWC) and non-responder subdivided into potential responder (true score change above SWC, below CI) such as non-responder (true change score CI below SWC). All statistical analyses were calculated using SPSS software (SPSS 22 inc./IBM, Armonk, NY, USA). 

Sample size was calculated via statistical power calculation (G*power 3.1.9.2, Düsseldorf, North Rhine-Westphalia, Germany)) on a medium effect size (f = 0.25), assuming *α* level of 0.05 and a desired power (1 − *β*) of 0.80 on group level. Thus, a sample size of *N* = 39 was determined. 

## 3. Results

### 3.1. Demographic Data

Table 1 provides detailed demographic data for the subjects. One-way ANOVA revealed no significant group differences for demographic data.

### 3.2. Baseline (t0)

**Figure 3:**
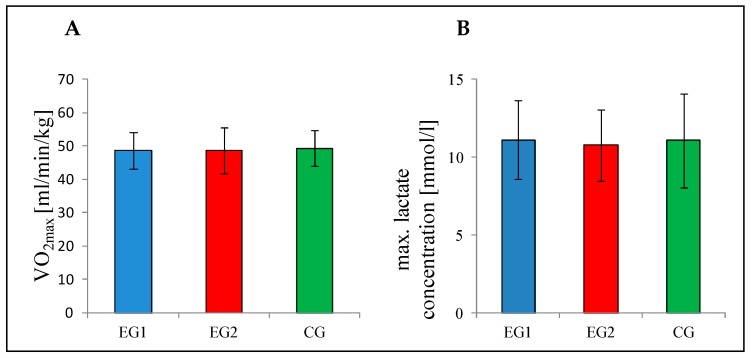
Parameters of the incremental step test at baseline. (**A**) Maximal oxygen uptake (VO_2max_) as a parameter for individual cardiovascular fitness and (**B**) max. lactate concentration (mmol/L) as a parameter for metabolic response. EG1 (experimental group 1, *n* = 13), EG2 (experimental group 2, *n* = 13), CG (control group, *n* = 13). Error bars represents the mean ± one standard deviation.

The three groups did not differ significantly with respect to the parameters of the incremental step test VO_2max_ (*F*_(2,36)_ = 0.05, *p* = 0.955), maximal amount of blood lactate levels (*F*_(2,36)_ = 0.06, *p* = 0.943) (Figure 3) and maximal heart rate (*F*_(2,36)_ = 0.93, *p* = 0.403). Additionally, one-way ANOVA revealed no significant group differences on any cognitive test variable at baseline (t0). 

### 3.3. Group Analysis

**Figure 4:**
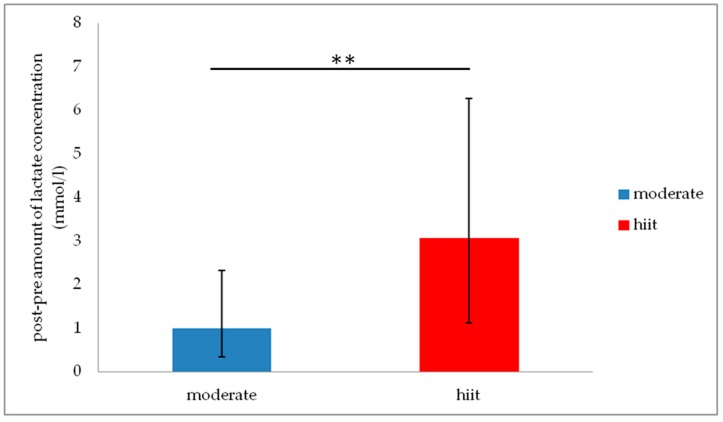
Amount of the blood lactate concentration post-pre-value (mmol/L) for moderate (*n* = 26) and hiit (*n* = 26) exercise condition, ** *p* < 0.001. Error bars represents the mean ± one standard deviation.

On group level, two-way repeated measures ANOVA revealed no significant main effect of group or group × time interaction on any cognitive test variable or the cognitive composite score in speed. A significant main effect of time was observed for the reaction time in Stroop task reading congruent condition (*F*_(1.62,56.70)_ = 31.72, *p* < 0.001, *η*^2^ = 0.48), TMT part A (*F*_(1.40,47.60)_ = 6.18, *p* = 0.009, *η*^2^ = 0.15), TMT part B (*F*_(1.60,54.48)_ = 17.94, *p* < 0.001, *η*^2^ = 0.35) and TMT B/A (*F*_(1.9,71.3)_, *p* = 0.013, *η*^2^ *=* 0.11) such as for the BZO score (d2-R) (*F*_(1.55,54.20)_ = 118.79, *p* < 0.001, *η*^2^ = 0.77). Post hoc tests showed significant decrease in reaction time in the Stroop task reading condition from t0 to t1(*p* < 0.001) and t0 to t2 (*p* < 0.001), in TMT part A from t0 to t1 (*p* < 0.001) and t0 to t2 (*p* = 0.046) such as in TMT part B from t0 to t1 (*p* = 0.001) and t0 to t2 (*p* < 0.001), as well as a significant increase in BZO score from t0 to t1 (*p* < 0.001), t1 to t2 (*p* < 0.001) and t0 to t2 (*p* < 0.001). Furthermore, accuracy revealed no main effect of group or group × time interaction for any cognitive test variable or the cognitive composite score. A significant main effect of time in accuracy was shown for Stroop task reading incongruent (*F*_(2,72)_ = 4.72, *p* = 0.012, *η*^2^ *=* 0.12) and naming congruent (*F*_(2,7)_ = 774, *p* = 0.001, *η*^2^ *= 0.17*) such as F% (*F*_(2,72)_ = 27.85, *p* < 0.001, *η*^2^ = 0.44). Post hoc tests revealed increased accuracy in Stroop task reading incongruent from t0 to t2 (*p* = 0.11), in naming congruent from t0 to t2 (*p* = 0.002) such as from t1 to t2 (*p* = 0.013) and in F% from t0 to t2 (*p* < 0.001). Moreover, t-tests showed no significant differences in the amount of the maximal blood lactate concentration between both experimental groups in as well the moderate (*t* = 0.53, *p* = 0.958) as the hiit (*t* = −1.10, *p* = 0.282) condition. Figure 4 shows a significant difference in the overall amount of the blood lactate concentration (post-pre-value) between moderate (*n* = 26) and hiit (*n* = 26) condition (*t* = −4.64, *p* < 0.001). Furthermore, no significant correlation between any exercise condition and a cognitive test variable such as the cognitive composite score was observed. 

### 3.4. Median Split

**Figure 5:**
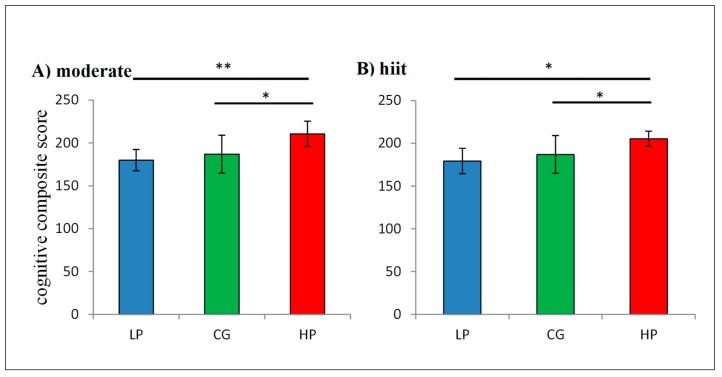
Post-hoc tests for cognitive composite score (**A**): moderate condition, (**B**): hiit condition. LCP = low cognitive performance (*n* = 13), HCP = high cognitive performance (*n* = 13), CG = control group (*n* = 13). A positive value reflects greater cognitive performance. * *p* < 0.05, ** *p* < 0.001. Error bars represents the mean ± one standard deviation.

Using a median split regarding low cognitive performance (LCP) and high cognitive performance (HCP) of the experimental group such as control group (CG) as reference, one-way ANOVA showed no significant differences regarding demographic data. Furthermore, one-way ANOVA showed significant differences in the moderate (*p* < 0.001) and hiit condition (*p* = 0.001) for the cognitive composite score (CCS). Additionally, Bonferroni post-hoc tests (Figure 5) revealed significant differences between HCP and CG for both moderate (*p* = 0.003) and hiit condition (*p* = 0.020). 

**Figure 6:**
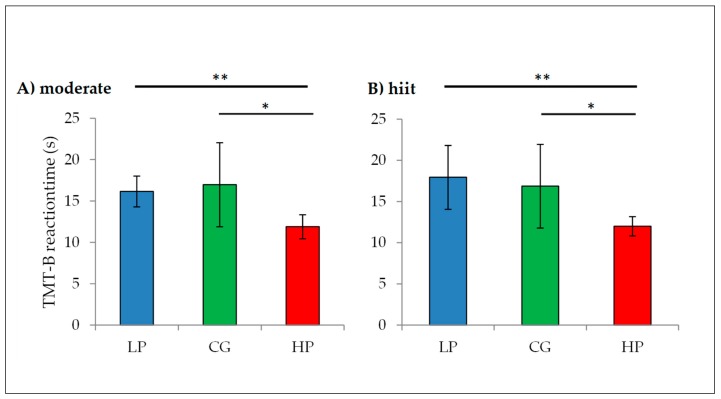
Post-hoc tests for TMT-B exemplary (reaction time in seconds). (**A**): moderate condition, (**B**): hiit condition. LCP = low cognitive performance (*n* = 13), HCP = high cognitive performance (*n* = 13), CG = control group (*n* = 13). Lower reaction time reflects greater cognitive performance. * *p* < 0.05, ** *p* < 0.001. Error bars represents the mean ± one standard deviation.

In addition, one-way ANOVA showed significant differences (*p* < 0.001) for the remaining test variables. Post-hoc comparisons (Figure 6 exemplary for TMT-B) regarding low cognitive performance and the control group showed significant differences in the Stroop naming interference condition (*p* = 0.027) and BZO (*p* = 0.022) such as between high cognitive performance and control group in BZO (*p* = 0,27), F% (*p* = 0.009) and TMT part B (*p* = 0.011) for the moderate condition. For the hiit condition post-hoc test detected significant differences between low cognitive performance and control group in BZO (*p* = 0.035) and between high cognitive performance and control group in BZO (*p* = 0.008), F% (*p* = 0.012), TMT part A (*p* = 0.018) and part B (*p* = 0.13). 

### 3.5. Intra-Individual and Inter-Individual Changes

**Figure 7:**
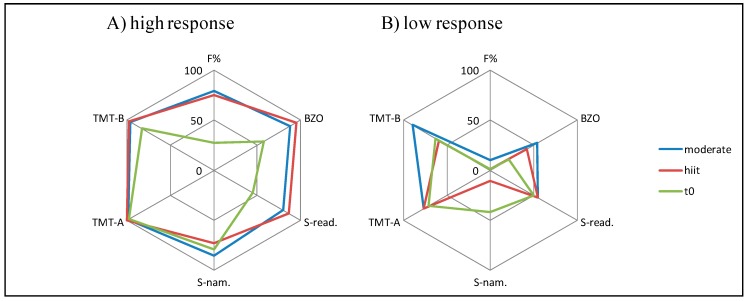
Cognitive performance (percentile rank) for baseline and after each intensity. Exemplary for a high cognitive performer (**A**) and a low cognitive performer (**B**) of the experimental group. Cognitive test variables including d2-R (F%, BZO), Stroop task reading condition (S-read.) and naming condition (S-nam.) and TMT (TMT-A, TMT-B). Green line: t0 (baseline), blue line: moderate condition, red line: hiit condition. The higher the net the higher the cognitive performance.

In contrast, intra-individual and inter-individual differences were achieved in several test scores (indicated in percentile range). Figure 7 shows exemplary intra-individual and inter-individual differences for two subjects of the experimental group. 

**Figure 8:**
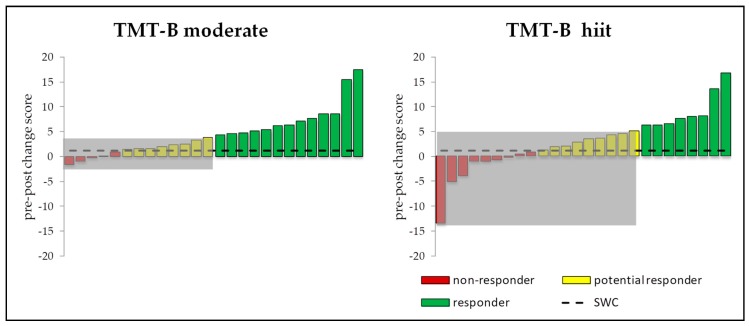
Individual response calculated with SWC (0.2 × between-standard deviation) and 50% *CI* (pre-post change ± typical error) for all subjects of the experimental groups (*n* = 26) for TMT-B moderate (left) and hiit (right) condition. Green bars labels responder (true score change *CI* above SWC), the grey area marks non-responder. The other subjects are divided into potential responder with true score change above SWC but below *CI* (yellow bars) and non-responder with true change score *CI* below SWC (red bars). Positive value reflects an increase of cognitive performance, a negative value vice versa.

Furthermore, Levene’s test showed only significant differences for TMT-B in the moderate (*p* = 0.013) and hiit (*p* = 0.034) acute exercise condition. Accordingly, using the SWC and the associated *CI* 13 subjects in the moderate and 8 subjects in the hiit condition showed a meaningful change and consequently were classified as responders (Figure 8). In contrast, using the same advance for the control group, only 3 subjects showed a meaningful change.

## 4. Discussion

Physical activity has several positive effects on cognition across the lifespan [24]. So far, most studies have focused on the effects of long-term exercise on structural and functional brain plasticity and cognition. However, a growing number of studies has investigated the effects of a single bout of exercise (acute exercise). In this context, a recent meta-analyses revealed small positive effects (*d* = 0.097; *n* = 1034) on cognitive functions (particularly executive functions) [6]. However, the included studies reported highly variable effects of a single bout of exercise on cognition, ranging from strongly positive to detrimental. A potential explanation could be, on the one hand, the differences in the employed exercise regimes (e.g., type of exercise, intensity, duration) and, on the other hand, the differences in neuropsychological assessments (e.g., lacking validation and reliability) or methodological limitations regarding the experimental design (e.g., missing control group or pretest) [7].

In our study using repeated measures ANOVA we could not find any significant main effect of group or group × time interaction neither for the cognitive composite score nor for any other cognitive test variable. However, our results replicate the non-significant effect on accuracy [8]. Moreover, some other studies also failed to observe significant effects regarding speed, or even found detrimental effects [25,26,27]. One potential reason for this null-effect could be our small sample size or the time point when the behavioural measures were tested with regard to the preceding acute exercise. In their meta-analysis on acute exercise studies (*n* = 40) Lambourne and Tomporowski reported detrimental effects on cognition when testing was performed during the exercise (*d* = −0.14) but improved cognition when subjects underwent testing after the exercise had been accomplished (*d* = 0.20) [28]. Several studies reported strong effects of acute exercise on executive functions; these lasted for up to two hours after exercise cessation [29,30]. 

In accordance with other studies our results do not support the postulated inverted-U-shape relationship between intensity and performance [6]. However, the results of the median split for each exercise condition and cognitive test variable provide initial evidence for inter-individual differences in the cognitive response within the experimental group. Therefore, we calculated additional inter-individual analyses. 

### 4.1. Responders vs. Non-Responders

Several exercise trials have presented an inter-individual variability in the response to physical exercise [17,31]. For example, changes in VO_2max_ have been shown to vary greatly (e.g., 0–100%) following a regular aerobic exercise [17,32]. Other metabolic (e.g., insulin sensitivity, cholesterol) and cardiorespiratory (e.g., blood pressure, heart rate at work load) parameters have shown inter-individually varying responses to physical exercise as well [17]. Based on these observations humans can be divided into responders and non-responders using a pre-determined threshold [33,34]. Currently, there is no gold-standard in exercise science to detect and distinguish responders and non-responders. Actual approaches using (i) a pre-determined threshold reflecting the typical error multiplied by two (2 × TE) [34,35], (ii) the calculation of the smallest worthwhile changes (SWC) based on 0.2 times standard deviation of baseline measurements [36] and/or (iii) the confidence intervals (CI) [33,37]. In our study we estimated the variability caused by the intervention for each test variable and consequently ran individual analysis only if significant differences in the variances between the experimental groups and the control group was shown. On this occasion, only TMT-B was admitted for further inter-individual analysis. Meaningful changes were calculated using 0.2 SWC and the associated CI for each subject [37]. In this way, the total number of identified responder (observed score change ± typical error beyond SWC) was *n* = 13 in the moderate and *n* = 8 in the hiit condition. Interestingly, in contrast to the group comparisons individual analysis supported some evidence for an inverted U-shape hypothesis between intensity and performance [6]. However, this relationship was only identified for TMT-B. Based on these observations, we can recommend to (i) apply physical exercise as a clinically meaningful intervention for cognitive enhancement for responders and (ii) identify optimal training modalities (moderate or hiit) to maximize the effects of physical exercise on cognition. However, this does not mean that non-responders should avoid physical exercise because of missing positive acute effects on cognition in general. Future research should consider multiple exercise protocols and derived recommendations for apparent non-responders.

### 4.2. Neurobiological Mechanisms of Exercise Induced Cognitive Enhancement

Up to now, the exact neurobiological mechanisms of how exercise induces cognitive enhancement is still largely unknown. Commonly discussed mediators are neurotrophic factors, especially the brain-derived neurotrophic factor (BDNF), which are well known for increasing adult neurogenesis, synaptic plasticity and memory [38]. BDNF can pass the blood brain barrier so that the peripheral BDNF concentration (in the blood) reflects the BDNF concentration in the brain [39,40]. While human studies have yielded robust evidence for an increase in peripheral BDNF concentrations after long-term physical exercise [41,42,43], less evidence exists for a single session of physical exercise [44]. Additionally, studies have shown a positive correlation between increasing BDNF concentration and improving memory [45] or executive control [46]. Furthermore, exercise intensity and the elicited blood lactate concentration seem to play an essential role in the exercise–cognition relationship. Lactate can also pass the blood brain barrier and function as an energy source, in addition to glucose [47]. Hayek and colleagues [48] found evidence that the exercise induced increase of lactate concentration in mice enhances the central BDNF signalling in hippocampus and improves learning and memory retention. In according with this results, in a human study Ferris, Williams and Shen [49] reported a positive correlation between the peripheral blood lactate and BDNF concentration for a single session of high intensity exercise (10% above ventilatory threshold) only, but not for a moderate intensity (20% below ventilatory threshold). Also, Schiffer and colleagues [50] found evidence that lactate per se and no other mechanism influences the regulation of BDNF blood concentration. Eight male subjects received an incremental intravenous infusion at rest with a four-molar sodium-lactate solution (infusion rate of 0.01 mL/kg/min every three minutes). The associated blood lactate concentration (comparable to high intensity exercise without acidosis) and the blood BDNF concentration were significantly increased after the infusion and decreased to baseline levels during the post-time (up to 60-min) [50]. For high exercise intensities the metabolic processes in the brain switch from glucose to lactate [51,52]. A recent meta-analysis reported that peripheral blood lactate concentrations above 2 mmol/L are necessary to stimulate the central lactate metabolism and associated signal cascades [53]. However, our results have shown significant group differences between moderate and high intensity exercise on blood lactate concentration, but no significant group effect nor group × time interaction effect for any cognitive test variable or the cognitive composite score. Thus, an influence of lactate on cognition following a single bout of exercise could not be detected. 

### 4.3. Limitations

This randomized controlled cross-over study has several limitations. First, the sample size was too small (*N* = 39) especially for the individual analysis. Using G*Power a sensitivity analysis with *α* level of 0.05, a desired power (1 − *β*) of 0.80 showed a ratio variance EG/variance CG of 5.395. Therefore, future research should examine the potential inter-individual variability in larger multicentre studies and/or using Bayesian statistics. Second, the neuropsychological test battery was probably too time consuming (30 min) so that possible effects may have been missed in the later tests. Additionally, motivation may have decreased due to the repeated cognitive assessments (three sessions), and/or the varying testing time during the day may have yielded further unwanted variation. Moreover, we did not measure the peripheral concentration of BDNF so that we cannot assess a relationship between the peripheral lactate concentration induced by different exercise intensities and BDNF. Furthermore, our sample was biased (male sample, aged 19–30 years, students). In addition, we did not assessed nutritional habits. Another limitation of the inter-individual analysis in our experimental design is that only three time points were assessed. Furthermore, collecting repeated measurements for each intensity (moderate and hiit) could reduce the impact of measurement errors [54,55].

## 5. Conclusions

In contrast to the current meta-analysis by Chang and colleagues [6], on a group level we could not detect any significant effect of a single bout of physical exercise on cognition. However, using a median split and threshold-based classification we could identify responders and non-responders in TMT-B among our subjects. In line with Chang and colleagues [6] our study showed exercise induced benefits of executive functions with a trend for a stronger effect after a moderate exercise session. Furthermore, our findings suggest that future work should not only use group-based statistics but also innovative approaches to identifying responders and non-responders. With that, exercise interventions should be advanced towards a personalized medicine [56]. Importantly, a first step for the approval of responder vs. non-responder analysis should be developed. Therefore, the current literature recommends a statistical test regarding the equality of variances such as the Levine’s test [57] and/or the intervention response standard deviation [37]. To reduce the misclassification of an individual’s performance and the associated replication crisis, only test variables with significant different change score variability (between experimental and control group) should be included for further analysis [37,57]. 

## Figures and Tables

**Table 1:** Demographic data.

	EG1 (*n* = 13)		EG2 (*n* = 13)		CG (*n* = 13)			
	*M*	*SD*	*M*	*SD*	*M*	*SD*	*F*	*p*
**Demographic Data**								
age (year)	23.54	3.05	24.00	3.56	22.46	3.13	0.76	0.472
height (cm)	185	0.06	182	0.08	178	0.07	2.71	0.080
weight (kg)	82.15	7.48	82.31	9.53	77.08	10.55	1.34	0.275
BMI (kg/cm^2^)	24.03	1.62	24.86	1.86	24.14	2.25	0.71	0.500
BDI (score)	4.92	3.64	4.08	2.81	7.08	4.39	2.31	0.114
**Physical Activity per Week**								
count	2.08	0.49	2.15	0.69	1.85	0.80	0.74	0.486
duration (h)	3.54	1.22	3.46	1.13	3.41	1.39	0.16	0.850

Demographic and exercise data of the sample (*N* = 39) subdivided for the three groups: experimental group 1 (EG1, *n* = 13), experimental group 2 (EG2, *n* = 13) and control group (CG, *n* = 13).
